# Matters Arising: Is congenital anosmia protective for Parkinson’s disease triggered by pathogenic entrance through the nose?

**DOI:** 10.1038/s41531-023-00538-5

**Published:** 2023-06-19

**Authors:** Alexander Wieck Fjaeldstad

**Affiliations:** 1 Department of Otorhinolaryngology, Flavour Clinic, University Clinic for Flavour, Balance and Sleep, Regional Hospital Gødstrup, Hospitalsparken 15, 7400 Herning, Denmark; 2 Flavour Institute, Department of Clinical Medicine, Aarhus University, Palle Juul-Jensens Boulevard 99, 8200 Aarhus N, Denmark

**Keywords:** Pathogenesis, Risk factors, Parkinson's disease

**arising from** Artin Arshamian et al. *npjParkinsonsDisease* 10.1038/s41531-022-00425-5 (2022)

In the recent commentary article, Arshamian et al.^1^ presented an interesting approach to investigate the theory that the olfactory bulb (OB) serves as the cradle of Parkinson’s Disease (PD) pathology. The authors hypothesised that the identification of a PD patient with Isolated Congenital Anosmia (ICA) and aplastic OBs would provide the basis for the falsification of the theory. With the low prevalence of ICA (1/10,000)^2,3^, such a patient would prove difficult to find, which leads the authors to raise awareness among clinicians to identify such a patient—a black swan. Here, we discuss the consequences of identifying a patient with PD and ICA and possible pitfalls in the interpretation of such a potential finding.

While the title suggests the quest for identifying a protective effect of ICA on the development of PD, the main hypothesis of the manuscript is that patients with ICA are immune to PD. The authors state that identifying a patient with ICA and PD would serve as a black swan to “…falsify the specific hypothesis that the OB serves as an initial staging area through which the disease propagates”^1^.

As such, there is a large discrepancy between the title and this statement, as the protective effects of ICA on PD are dissimilar to the hypothesis of PD immunity in ICA patients. The identification of an ICA-PD-black swan does not rule out that ICA may have a protective effect on PD in general. This would fall in line with prior studies on the vagus nerve - the only other cranial nerve with exposure to external pathogens and first synapse in the brain or brainstem—where the protective effect of vagotomy on PD development has been suggested^4^.

An ICA-PD-black swan would not falsify that the “…OB serves as an initial staging area through which the disease propagates”, but rather falsify that the OB serves as THE ONLY initial staging area through which the disease propagates. This emphasises the relevance of an ICA-PD-black swan as suggested by Arshamian et al.^1^, as such a patient would give rise to a falsification of the ‘Dual-Hit’ theory, as the pathological process does not have to occur at two sites simultaneously (anterogradely, via olfactory pathways; and retrogradely, via preganglionic vagal fibres and enteric plexuses)^5^. Contrary, it would support the theory highlighted in the recent study by Borghammer et al. that Lewy pathology may take two distinct independent routes resulting in either gut-first PD or brain-first PD^6^.

If the quest for a black swan succeeds, several pitfalls may emerge. The characterisation of the PD subtype must have the utmost focus as this may affect the generalisability and relevance of the case. Furthermore, anatomical abnormalities of olfactory pathways similarly complicate any absolute falsification of theories on the possible avenues of Lewy pathology propagation. This is exemplified by another black swan in olfactory literature; a patient with normal olfactory function and absence of olfactory bulbs has recently been identified^7^. Although not fully understood, a few more similar patients without OBs have been identified with cortical activation of olfactory stimuli and olfactory function within the normal range in psychophysical olfactory testing. Albeit rare, this phenomenon with unchartered olfactory pathways would necessitate prudence regarding absolute conclusions if an ICA-PD-black swan is identified; the ICA diagnosis is based on the patient’s reported history with no recollection of ever being able to smell^3^ and aplastic/absent olfactory bulbs on MRI. As such, a clinician with thorough experience in diagnosing congenital olfactory disorders is essential and an extended battery of olfactory electrophysical testing and neuroimaging would be warranted.

Even with the discovery of an ICA-PD-black swan, further studies to investigate the protective effects of olfactory disorders on PD are needed, such as case-control studies on normosmic controls and patients with ICA (and/or syndromic anosmia and/or anosmia caused by head trauma early in life).

In conclusion, identifying an ICA-PD-black swan patient is highly relevant, as reported by Arshamian et al.^1^ While such a case with thorough clinical confirmation of the ICA and PD diagnosis neither falsifies the theory of ICA being protective of PD nor quantifies any potential protective effects of ICA on PD, it does falsify the simultaneous ‘Dual-Hit’ theory and increase the likelihood of other theories such as the separate entity of ‘gut-first’ and ‘brain-first’ PD.

## References

[1] Arshamian A, Iravani B, Lundström JN (2022). Is congenital anosmia protective for Parkinson’s disease triggered by pathogenic entrance through the nose?. npj Parkinsons Dis..

[2] Sailani MR (2017). Isolated congenital anosmia and CNGA2 mutation. Sci. Rep..

[3] Karstensen HG, Tommerup N (2012). Isolated and syndromic forms of congenital anosmia. Clin. Genet..

[4] Svensson E (2015). Vagotomy and subsequent risk of Parkinson’s disease. Ann. Neurol..

[5] Hawkes CH, Del Tredici K, Braak H (2007). Parkinson’s disease: a dual-hit hypothesis. Neuropathol. Appl. Neurobiol..

[6] Borghammer P (2022). A postmortem study suggests a revision of the dual-hit hypothesis of Parkinson’s disease. npj Parkinsons Dis..

[7] Weiss T (2020). Human olfaction without apparent olfactory bulbs. Neuron.

